# Our future

**DOI:** 10.1016/S1808-8694(15)31166-6

**Published:** 2015-10-19

**Authors:** João Ferreira de Mello

**Affiliations:** Editor - RBORL

Dear Colleagues,

The current editorial executive board is celebrating two yeas in office. On its behalf and as Chief Editor, I would like to share with you some information about our journal.

The major goal of the Brazilian Journal of Otorhinolaryngology is to convey scientific update to the members of the Brazilian Association of Otorhinolaryngology - Neck and Facial Surgery. Here we publish papers that innovate or enhance our knowledge basis on Otorhinolaryngology, concerning basic experimental research, unveiling physiology and/or physiopathology, diagnostic methods (clinicolaboratorial work up) or treatment (clinical or surgical) for the disorders associated with our specialty.

In these two years, 661 papers were submitted to our office. As we analyze the topics submitted, we see that otology and neurotology represented 36% of the total, followed by rhinology (21%), laryngology and voice (13%), pharyngology (11%), and the remaining 19% had to do with topics pertaining to head and neck surgery, pediatric otolaryngology, facial plastic surgery, skull base surgery, and others.

Our journal has an International Qualis C classification at Capes, and such status was attained thanks to the work done by the editors who preceded me and, obviously, the work of our authors. Our goal is to upgrade this classification, and to achieve it we count on a group of demanding and highly qualified colleagues - Associate Editors and Reviewers among them - who assess the manuscripts. Therefore, papers without proper scientific methodology and those which are not relevant to our specialty will not be approved for publication.

As to the time taken to assess and publish the manuscripts, we must stress that in 2006, it took approximately 122 days between paper submission and the final decision regarding its publication. This last year we managed to reduce this time to 74 days. Unfortunately - and for a number of reasons - the time it takes to publish a paper is still long, and this is due to numerous factors, especially the large number of papers submitted weekly to our office. In the case of original papers, this time can take up to one year, and even longer for case reports and review articles. Consequently, the large number of such papers we have in store made us temporarily suspend submissions until we publish all our stock of approved papers.

Some authors may be wondering why their papers are still waiting assessment, even those submitted a while ago. Please notice that the authors receive messages from the journal through e-mail; therefore, it is paramount to keep your e-mail addresses updated, otherwise you may miss some important communication from us. There are over 100 studies “seating” in our journal, which were sent back to the authors with the corrections requested by our editors and were not sent back to us. Soon, we will establish a due date for these papers to be corrected (or properly explained why they should remain the way they are) and sent back to us. After this period the papers will be automatically removed from the system.

To the authors who send papers, we kindly ask you to format and arrange them according to our submission criteria (listed in the journal's Web site), especially in regards of the references and the use of keywords based on the DeCS (Health Sciences Keywords) and MeSH (Medical Subject Headings), otherwise they will be refused for not matching our journal's standards. At the end of the submittal process, there will be a checklist in order to make sure the text is within our standards. Please notice that these measures are not to be taken as some form of punishment, but rather as a means of classification considering the international readership of our journal, which was attained thanks to the hard work of many people, which is extremely important for our association and for the graduate programs of the many institutions associated with our specialty.

Best Regards

